# The twisted structure of the fetal calcaneal tendon is already visible in the second trimester

**DOI:** 10.1007/s00276-020-02618-0

**Published:** 2020-11-25

**Authors:** Paweł Szaro, Grzegorz Witkowski, Bogdan Ciszek

**Affiliations:** 1 Department of Radiology, Institute of Clinical Sciences, Sahlgrenska Academy, University of Gothenburg, Göteborgsvägen 31, 431 80 Gothenburg, Sweden; 2 Department of Musculoskeletal Radiology, Sahlgrenska University Hospital, Gothenburg, Sweden; 3 Department of Descriptive and Clinical Anatomy, Medical University of Warsaw, Warsaw, Poland

**Keywords:** Calcaneal tendon, Fetus, Tendon, Gastrocnemius, Soleus, Development

## Abstract

**Introduction:**

The progress in morphological science results from the greater possibilities of intra-pubic diagnosis and treatment of congenital disabilities, including the motor system. However, the structure and macroscopic development of the calcaneal tendon have not been investigated in detail. Studies on the adult calcaneal tendon showed that the calcaneal tendon is composed of twisted subtendons. This study aimed to investigate the internal structure of the fetal calcaneal tendon in the second trimester.

**Materials and methods:**

Thirty-six fetuses fixed in 10% formaldehyde were dissected using the layer-by-layer method and a surgical microscope.

**Results:**

The twisted structure of the calcaneal tendon was revealed in all specimens. The posterior layer of the calcaneal tendon is formed by the subtendon from the medial head of the gastrocnemius muscle. In contrast, the anterior layer is formed by the subtendon from the lateral head of the gastrocnemius muscle. The subtendon from the soleus muscle constitutes the anteromedial outline of the calcaneal tendon. The lateral outline of the calcaneal tendon is formed by the subtendon originating from the medial head of the gastrocnemius muscle. In contrast, the medial outline is formed by the subtendon from the soleus muscle. In most of the examined limbs, the plantaris tendon attached to the tuber calcanei was not directly connected to the calcaneal tendon.

**Conclusions:**

The twisted structure of the subtendons of the fetal calcaneal tendon is already visible in the second trimester and is similar to that seen in adults.

## Introduction

Fetal diagnostics are developing that add to the progress in morphological knowledge. However, the structure of the fetal calcaneal tendon has not been revealed. Anatomic knowledge of the locomotor system is essential to diagnose congenital diseases and discover advanced intrauterine operation methods. Rotation of the calcaneal tendon fibers influences surgical techniques for lengthening the calcaneal tendon, which can be indicated in more than 85% of cases [14, 17]. Failure of the surgery may result in balance difficulties or hyperextension of the knee joint, leading to further motor disability and deformations [13, 17]. In contrast to the adult calcaneal tendon, the twisted structure of the calcaneal tendon in fetuses has not been investigated in detail. The aponeuroses located on the surfaces of the triceps surae muscle extend downward, forming twisted subtendons [33, 35]. In the soleus muscle, the aponeurosis joins anteriorly the inconsistent central tendon, significantly increasing the area of muscle fiber attachment [2]. The three-dimensional aponeurosis structure enables a multi-pennate muscle arrangement increasing the energy efficiency of the muscle.

Three subtendons constitute the adult calcaneal tendon [10, 21, 35]: the subtendon from the medial head of the gastrocnemius muscle (S-MGC) forms the posterior layer, the subtendon from the lateral head of the gastrocnemius muscle (S-LGC) constitutes the anterior layer and the subtendon of the soleus muscle (S-Sol) forms the medial outline. In contrast, the lateral outline is formed by the subtendon from the medial head of the gastrocnemius muscle [21, 35]. In the calcaneal tendon, the gastrocnemius and soleus muscles differ functionally, therefore knowledge of the internal structure can improve function restoration after trauma or during correction of congenital deformities.

The strain of each of the subtendon of the calcaneal tendon is not constant. Noteworthy differences in tension are noticed mostly in the tendons with a least or extreme degree of torsion. Heterogeneous tendon tension can increase the risk of developing calcaneal tendon disorders [12].

The presence of the twisted structure of the calcaneal tendon probably has a developmental and biomechanical background. Rotation of the lower limb’s bud can be a developmental factor that influences the calcaneal tendon’s internal arrangement [13, 28, 30]. However, both the tendon and the muscle originate from two different embryological origins but with common mesodermal compartments [36]. Experiments on animal embryos showed that development of the tendon could be independent of expansion of the muscle belly. Even so, the role of the tendon primordium is vital for differentiation of the skeletal muscle primordium. The final normal tendon maturation also requires the presence of the muscle belly [3, 16, 27]. The primordium of the triceps surae muscle is located firstly on the lateral side of the calf, together with the common flexor muscle mass. At this developmental stage, the bellies of the gastrocnemius muscle are indistinguishable [3, 16]. Due to stimulation by growth of the calcaneal tendon primordium, the gastrocnemius origins migrate to its adult localizations. However, there are no precise data on this developmental stage [16]. Developmental disorders of calcaneus position may influence the degree of pelvic anteversion and load on the lower extremity. Thus, biomechanics of the subtalar joint and the calcaneal tendon can be impaired [12].

To our knowledge, there are no studies on the arrangement of subtendons in the calcaneal tendon in the early developmental stages. This study aimed to describe the internal anatomy of the fetal calcaneal tendon in the second trimester.

## Materials and methods

This study was conducted on 36 fetuses (19 male and 17 female) from the collection of the Department of Clinical and Descriptive Anatomy of the Medical University of Warsaw, Poland. Fetuses were spontaneous abortions. All specimens were fixed in 2% glutaraldehyde and were placed in water for 24 h before dissection. The gestational age of specimens ranged from 13 to 22 weeks and was calculated from the crown–rump length, femur length, foot length, biparietal diameter and head circumference [26]. The material included specimens without external pathology of the limbs and anomalies of the central nervous system. Fetuses come from a voluntary donation program and are part of the existing collection of the Department of Clinical and Descriptive Anatomy, Medical University of Warsaw, Poland. The research was conducted according with the Polish Death and Funeral Act and with relevant guidelines and regulations. The Institutional Ethics Committee was informed about the ongoing study, and the Committee stated no need for its approval.

A layer-by-layer dissection method, microsurgical instruments and surgical microscope (magnification 4× and 10×) were used. Firstly, we identified aponeurosis which were defined as flat layer of fibro-tendinous tissue which serves as the site of attachment of muscle fibers. The aponeurosis on the anterior surface of the gastrocnemius heads and the posterior surface of the soleus extend downward, forming appropriate subtendons. Then, dissection of each subtendon continued distally to the tuber calcanei. Photographic documentation was made at each stage of dissection. The terminology of the internal structure of the calcaneal tendon is according to Handsfield et al. [15].

## Results

**Fig. 1:**
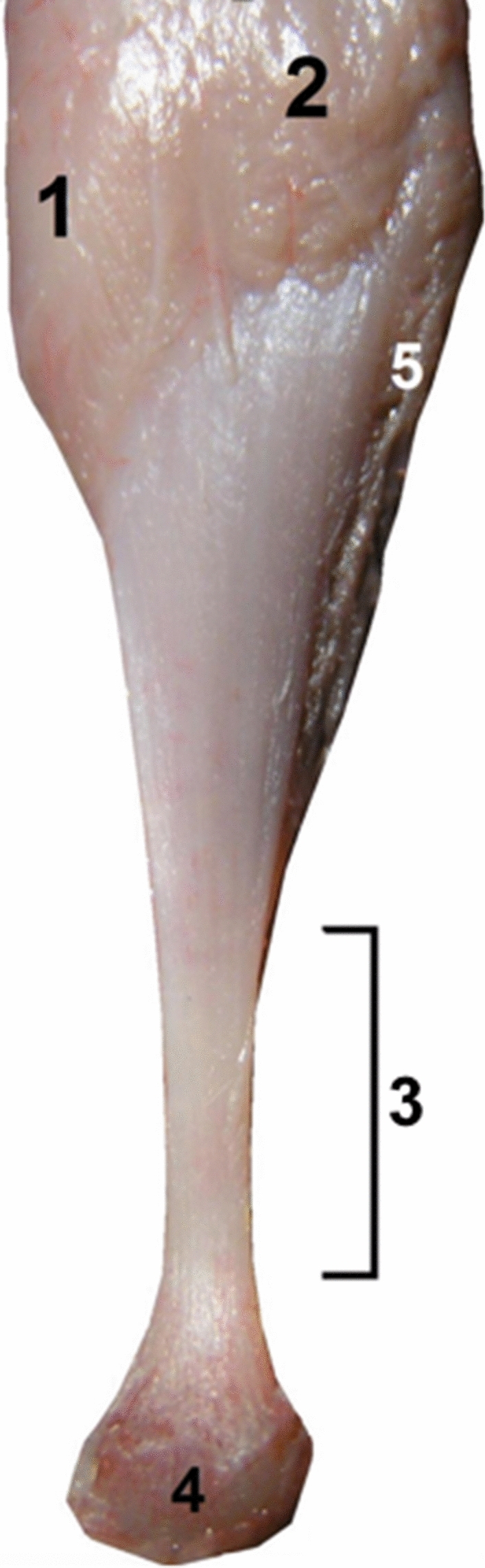
The right calcaneal tendon, posterior view, 18 weeks’ gestation: (1) the S-MGC; (2) the S-LGC; (3) the calcaneal tendon just above the tuber calcanei; (4) the tuber calcanei; (5) the S-Sol

**Fig. 2:**
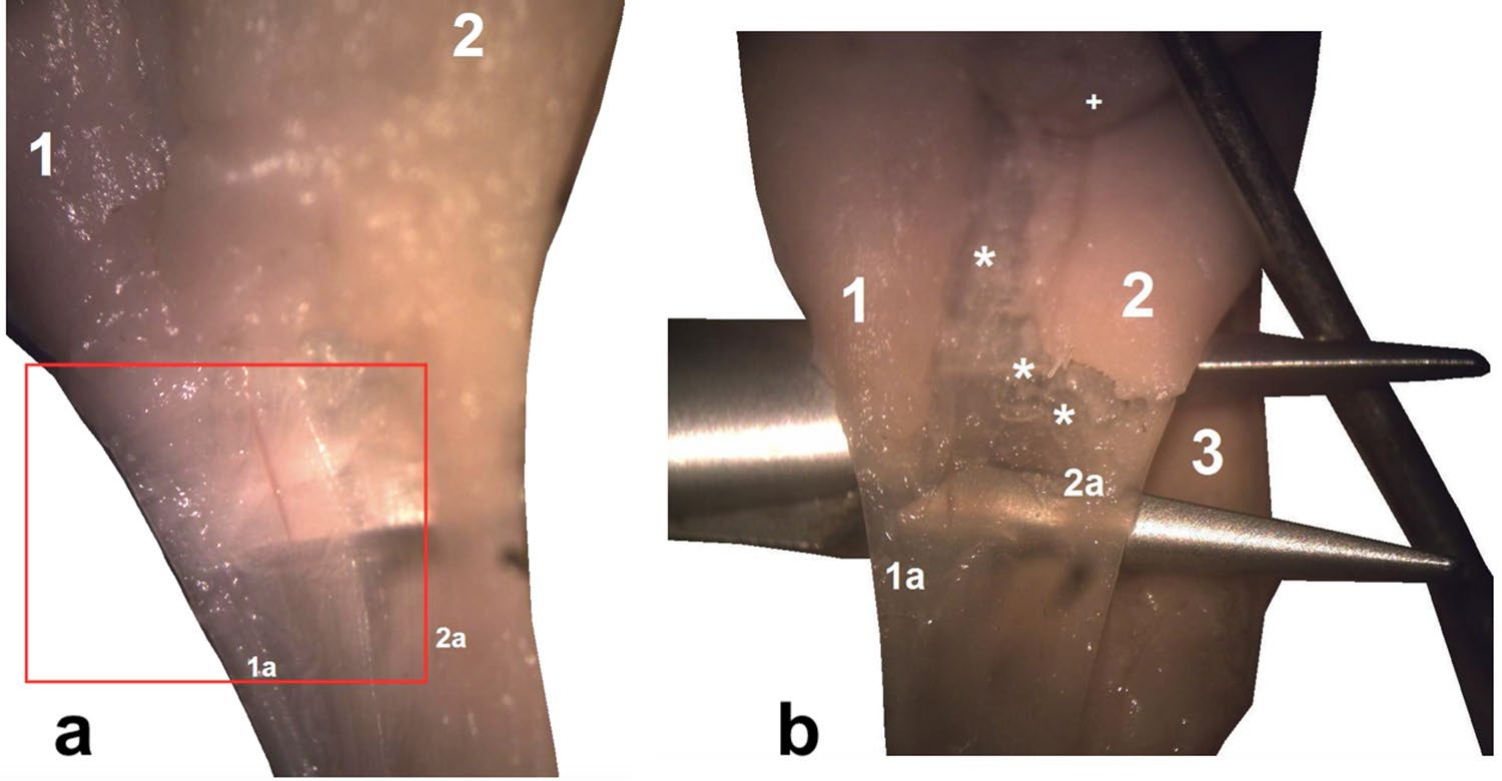
The aponeuroses and the calcaneal tendon (**a**, **b**: 13 weeks’ gestation): (1) the medial head of the gastrocnemius muscle; (1a) the S-MGC; (2) the lateral head of the gastrocnemius muscle; (2a) the S-LGC; (3) the soleus muscle; (3a) the S-Sol. The asterisk (*) indicates the deep aponeurosis of the gastrocnemius muscle, which is located on the anterior outline of the muscle belly

Rotation of the subtendons was seen in all examined specimens. The most prominent rotation was in the midportion of the calcaneal tendon (Figs. 1 and 2). The three twisted subtendons are prolongations of the triceps surae aponeuroses (Fig. 2). The deep aponeurosis of the gastrocnemius and the superficial aponeurosis of the soleus unite in the lower half of the leg to form the calcaneal tendon (Figs. 2 and 4). The central tendon of the soleus was present in 66 cases (91.7%) and united with the posterior aponeurosis of the soleus muscle to form the calcaneal tendon.

All subtendons could be relatively easily dissected in the superior 1/3 of the calcaneal tendon, whereas, in the lower parts it was more challenging to separate them. The superior 1/3 of the calcaneal tendon reveals the same arrangement as aponeurosis of the triceps surae.

**Fig. 3:**
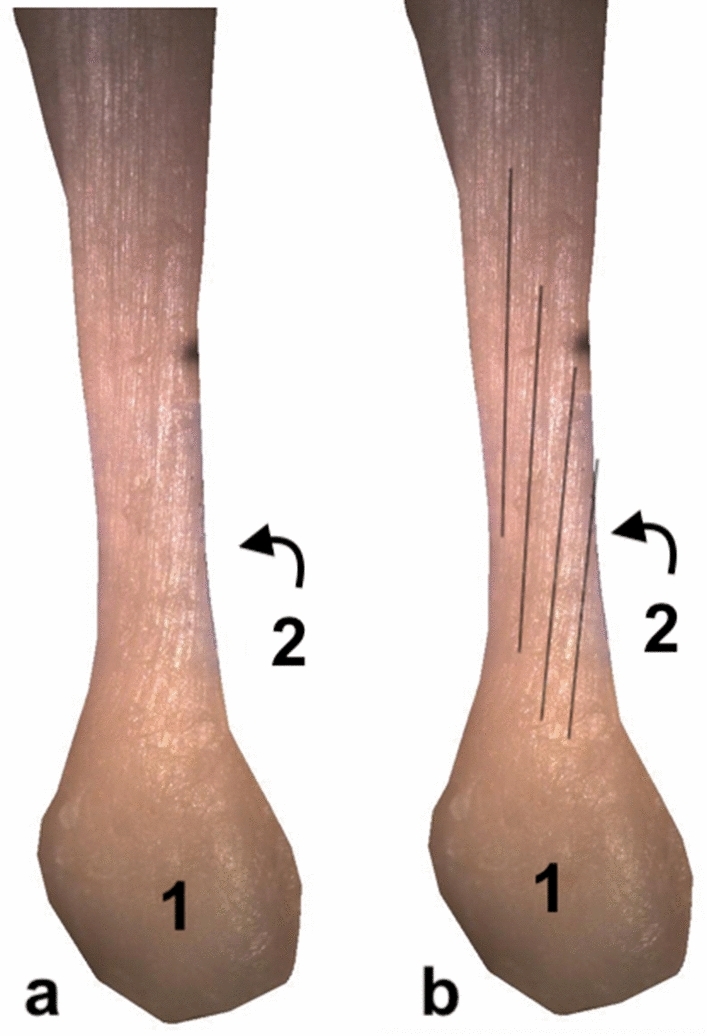
The left calcaneal tendon above the tuber calcanei: (1) posterior view, 14 weeks’ gestation; (2) the medial outline. Black lines mark the rotation of the fibers

**Fig. 4:**
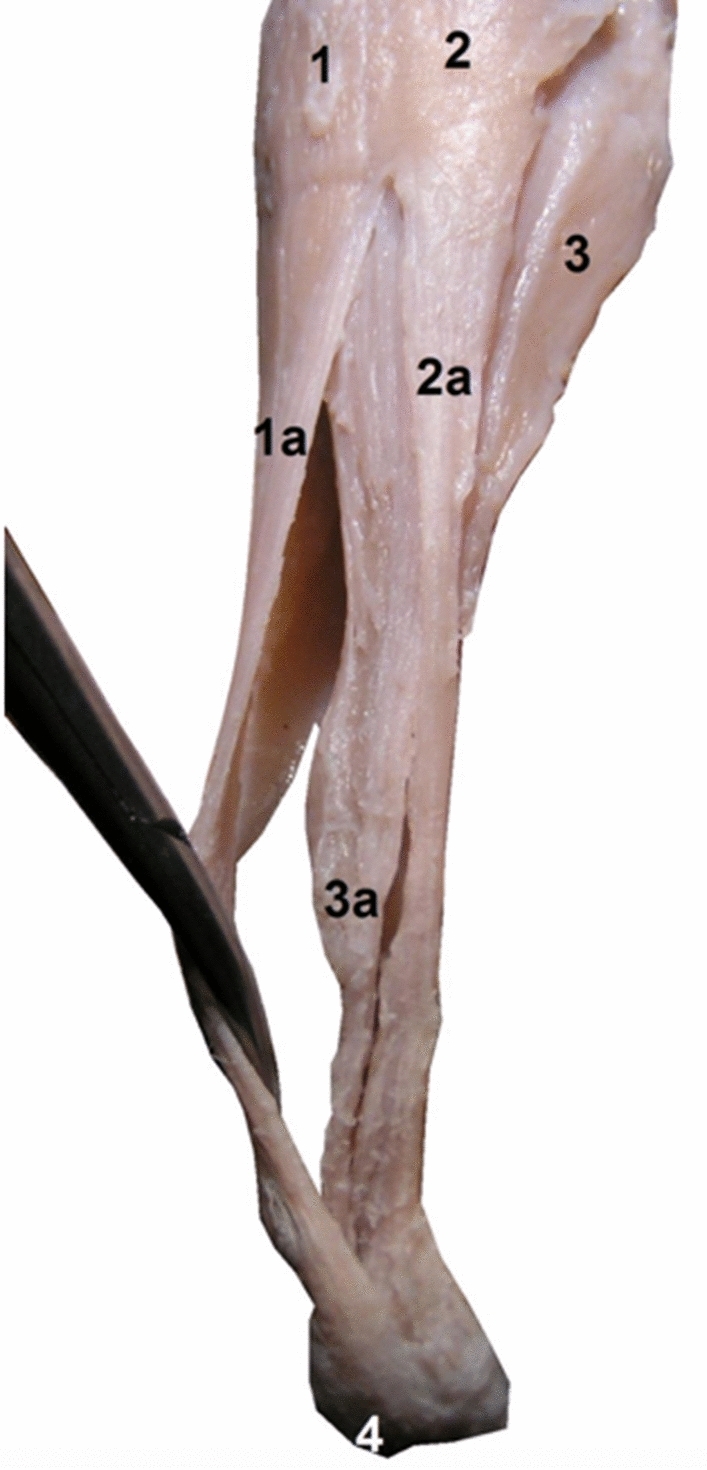
The right calcaneal tendon, posterior view, 19 weeks’ gestation: (1) medial head of the gastrocnemius muscle; (1a) the S-MGC; (2) lateral head of the gastrocnemius muscle; (2a) the S-LGC; (3) the soleus muscle; (3a) the S-Sol; (4) the tuber calcanei

**Fig. 5:**
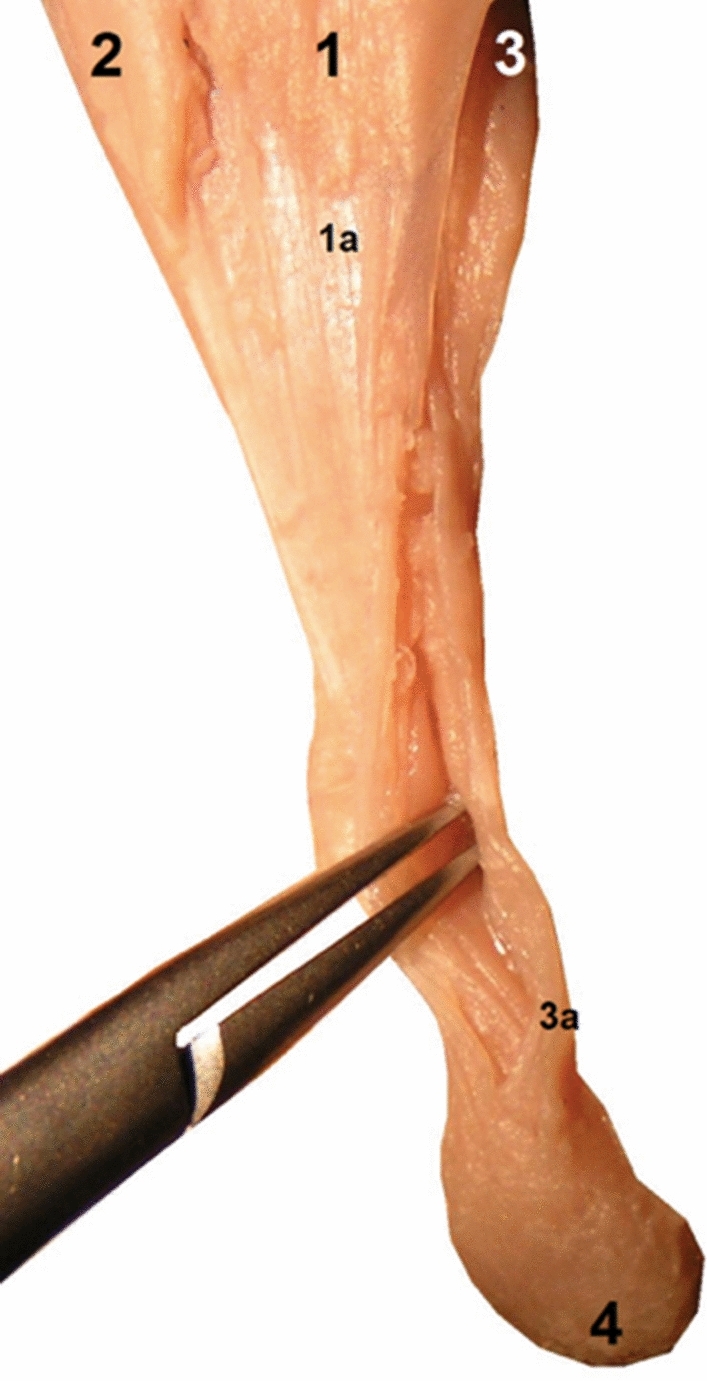
The left calcaneal tendon, posterior view, 15 weeks’ gestation: (1) the medial head of the gastrocnemius muscle; (1a) S-MGC; (2) the lateral head of the gastrocnemius muscle; (3) the soleus muscle; (3a) the S-Sol; (4) the tuber calcanei

**Fig. 6:**
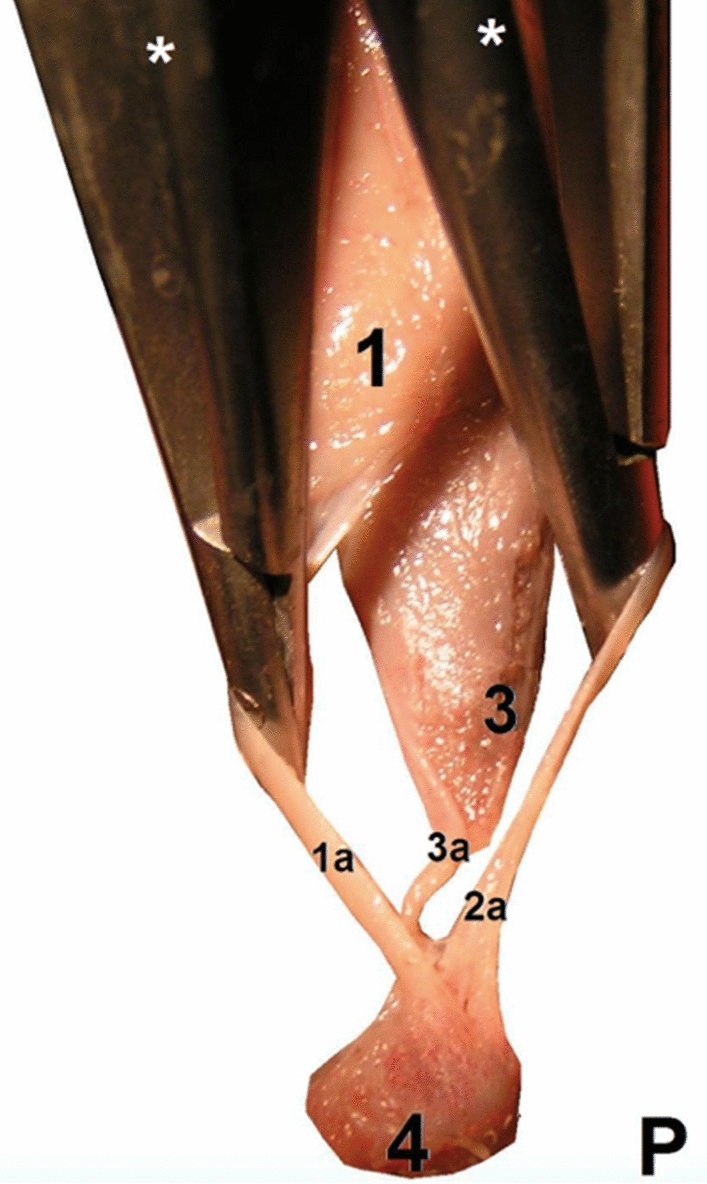
The right calcaneal tendon, posterior view, 13 weeks’ gestation: (1) medial head of the gastrocnemius muscle; (1a) the S-MGC; (2a) the S-LGC; (3) the soleus muscle; (3a) the S-Sol; (4) the tuber calcanei. The asterisk (*) indicates the surgical forceps

The S-LGC runs downward, anteriorly and medially, to form the anterior layer of the calcaneal tendon (Figs. 3, 4, 5). The S-MGC runs inferiorly, laterally and posteriorly to form the posterior layer of the calcaneal tendon. The S-Sol runs downward, reaching the medial and anterior part of the calcaneal tendon (Figs. 3, 4, 5, 6). The midportion and the insertion show the same arrangement of subtendons.

The anterior outline of the calcaneal tendon (Table 1, Fig. 6) is formed by the S-LGC and S-Sol: S-LGC dominance is seen in 72.2%, co-dominance of S-LGC and S-Sol in 25% and S-Sol dominance in 2.8%. In each case, the anterior outline of the calcaneal tendon was related to the macroscopically fully developed bursa and Kager’s fat pad.

**Table 1:** Contribution of the outlines of the calcaneal tendon (*n* = 72)

The outlines of the calcaneal tendon	*n*	%
Anterior outline		
Dominance of the S-LGC	52	72.2%
Co-dominance of the S-LGC and S-Sol	18	25.0%
Dominance of the S-Sol	2	2.8%
Posterior outline		
Only the S-MGC	68	94.4%
Dominance the S-MGC over S-Sol	4	5.6%
Medial outline		
Only the S-Sol	49	68.1%
Dominance of the S-Sol and S-MGC	20	27.8%
Co-dominance of the S-Sol and S-MGC	2	2.8%
Dominance of the S-MGC	1	1.4%
Lateral outline		
Only the S-LGC	1	1.4%
Dominance of the S-LGC	3	4.2%
Dominance of the S-MGC	11	15.3%
Only the S-MGC	57	79.2%

**Fig. 7:**
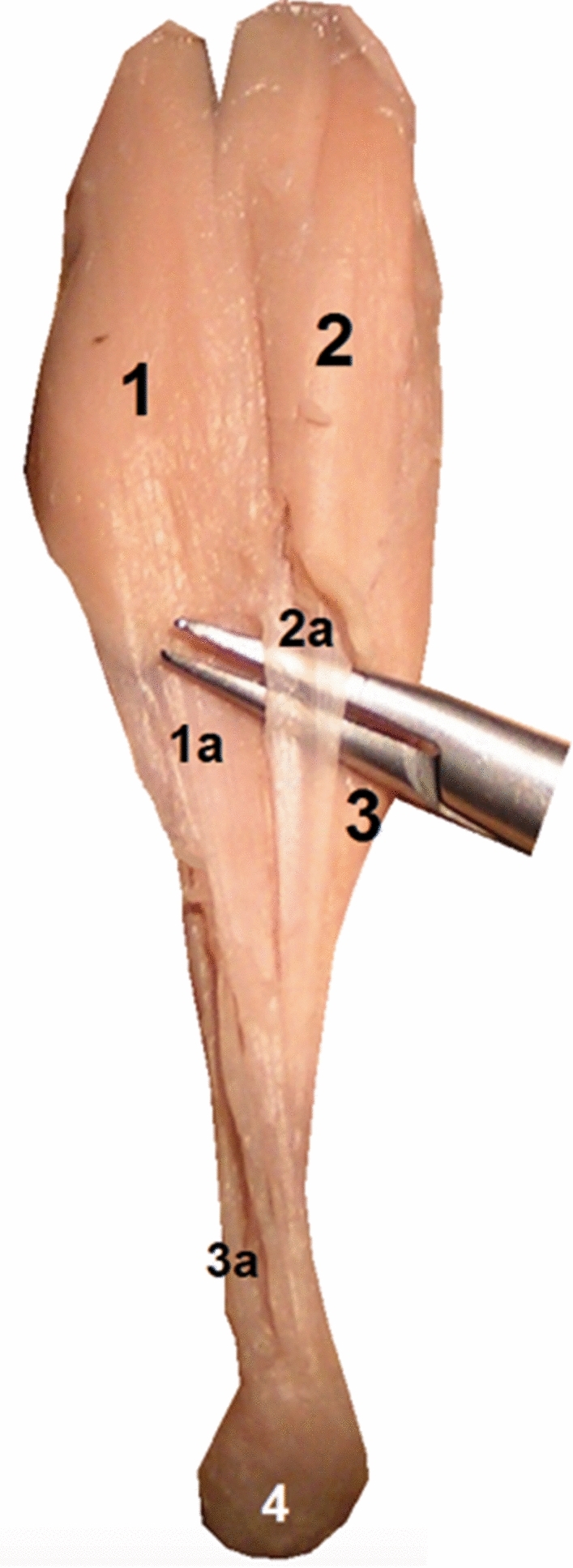
The right calcaneal tendon, posterior view, 15 weeks’ gestation: (1) the medial head of the gastrocnemius muscle; (1a) the S-MGC; (2) the lateral head of the gastrocnemius muscle; (2a) the S-LGC; (3) the soleus muscle; (3a) the S-Sol; (4) the tuber calcanei

The posterior outline of the calcaneal tendon (Table 1, Figs. 4, 5, 6, 7) is commonly composed only of the S-MGC (94.4%; Fig. 3); however, in 5.6% it is formed by the S-MGC and S-Sol, with S-MGC domination.

The medial outline of the calcaneal tendon (Table 1, Fig. 5) is formed most often only by the S-Sol (68.1%). Dominance of the S-Sol over S-MGC was seen in 27.8%. Other variants, such as co-domination of the S-Sol with S-MGC or dominance of the S-MGC, are rare and together constitute no more than 4.2% (Table 1).

**Fig. 8:**
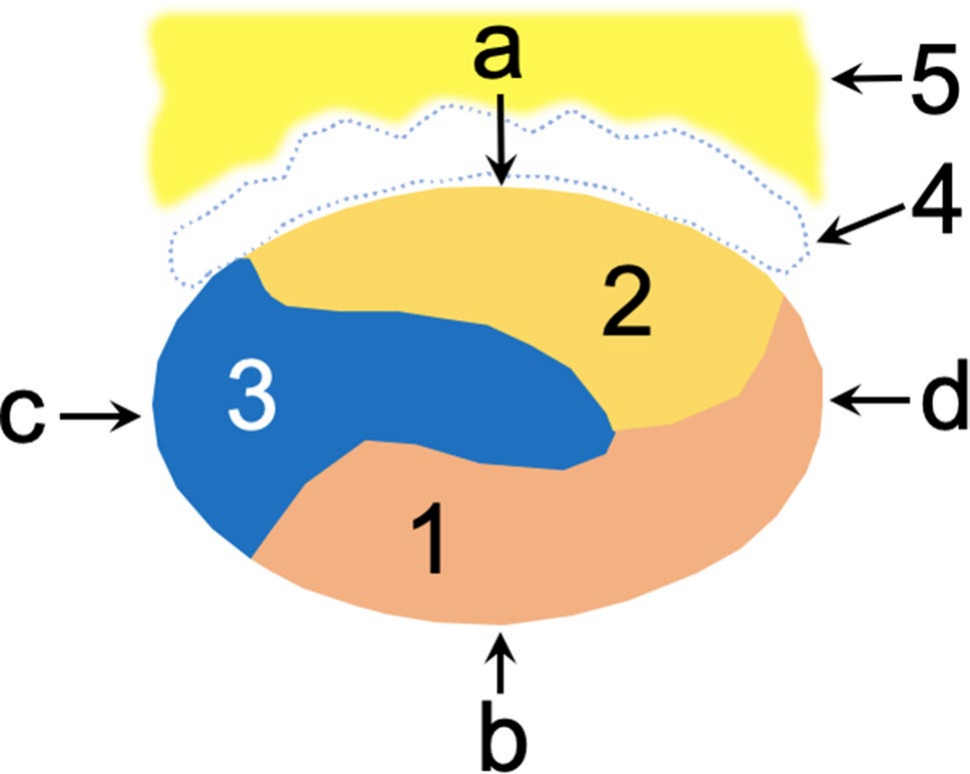
Horizontal section through the midportion of the fetal calcaneal tendon: (1) the S-MGC; (2) the S-LGC; (3) the S-Sol; (4) the deep calcaneal bursa; (5) Kager’s fat pad. The outlines of the calcaneal tendon: (**a**) anterior; (**b**) posterior; (**c**) medial; (**d**) lateral

The lateral outline of the calcaneal tendon (Table 1, Fig. 6) was formed only by the S-MGC in 79.2%. Dominance of the S-MGC over S-LGC was noticed in 15.3%. Occasionally, only the S-LCG or dominance of the S-LGC over S-MGC was seen (Fig. 8).

The plantaris muscle was present in 87.5% of the examined limbs (Table 2). We noticed that type 1 (30.6%) and type 2 (37.4%), according to Olewnik et al. 2017 [19], are the most common. Types 1, 2, 3 and 5 show direct contact with the medial outline of the calcaneal tendon (77.8%), whereas type 4 represents insertion anterior to the calcaneal tendon (9.7%).

**Table 2:** Types of plantaris tendon according to Olewnik et al. [19]

Type of plantaris tendon	Right	Left	Total	%
1	9	13	22	30.6%
2	13	12	25	34.7%
3	3	3	6	8.3%
4	4	3	7	9.7%
5	3	0	3	4.2%
Absent	6	3	9	12.5%

## Discussion

The twisted structure of the calcaneal tendon was shown to develop in the first trimester because the rotation of subtendons was visible in all the studied material. The S-Sol is located anteromedially. The S-LGC forms the anterior part and the S-MGC the posterior part of the calcaneal tendon. The spiral arrangement of fibers results in the S-MGC comprising most of the posterior outline of the calcaneal tendon. In contrast, the anterior outline is formed mainly by the S-LGC [10, 11, 13, 21, 35]. The twisted structure of the fetal calcaneal tendon observed in our study is similar to that described in adults [4, 10–13, 21, 24, 35]. Such an arrangement of fibers means that simple end-to-end connection of the calcaneal tendon will probably not fully restore its function [13, 14]. To our knowledge, the location of the subtendons in the fetal calcaneal tendon has not yet been studied but the spiral direction of fibers has been reported in newborns. The twisted arrangement of the subtendons in the calcaneal tendon revealed in our study is possibly an evolutionary solution, which is also seen in animals [9, 29, 34]; it may indicate an evolutionary and locomotive background because the spiral structure of the fibers is more effective at storing and releasing energy [22]. Based on our results, the development of the calcaneal tendon must start within the first trimester, probably similar to the anatomic maturation of the flexor tendon of the foot, which occurs earlier than the 8th week [6, 37]. Unfortunately, we did not find similar studies on the development of the calcaneal tendon.

There are similarities in the structure of the calcaneal tendon and its components in fetal life and after birth. Different tendon torsion causes variable tension, which may consequently increase the risk of pathological changes, especially in tendons with slight or significant torsion [12]. Except for the arrangement of the subtendons described above, the system of aponeurosis is similar. The individual subtendon is an extension of the aponeurosis to the calcaneal tendon, therefore in the upper 1/3 of the calcaneal tendon the subtendon system reflects localization of the muscle. Thus, the muscle belly, the aponeurosis and the tendon from a functional musculotendinous unit. Active tracking and dissection of the subtendons probably occurs along the thin membranes, which enable dissection to the calcaneus [23]. The membranes are present mostly in the midportion of the adult calcaneal tendon [1] and probably serve as space for the course of vessels and nerves [18].

In the insertion of the calcaneal tendon, unlike the midportion, the subtendons are tightly packed, which made the separation a little more complicated and it was necessary to use an operating microscope. Similar results were obtained in studies of the adult calcaneal tendon [35], which also confirms that development of the calcaneal tendon needs to occur within the first trimester of pregnancy.

Functional and structural changes to the calcaneal tendon, such as upper migration of the insertion, can be observed during life [20]. Macroscopic components of the adult enthesis organ [5] were identified in our study, possibly due to stimulation to maturity of the tendon primordium. A fully developed deep calcaneal bursa in fetuses was reported during the 9th week of development [28]. It is difficult to determine whether the fibrocartilage of the enthesis organ was present in fetuses because our study was not histological. There are some differences between the fetal and adult enthesis organ. In addition to the increased diameters of the deep calcaneal bursa, there is also an increasing protrusion of Kager’s fat pad in the deep calcaneal bursa, probably due to adaptation to movements and mutual compression between the tendon and the calcaneus [5, 25].

We believe that the results of our research may help to develop the new surgical techniques of calcaneal tendon lengthening. The rotation of the subtendons can increase the difficulty of percutaneous calcaneal tendon lengthening because rotation of tendon fibers makes it difficult to pin down the proportion in the cross-section of the calcaneal tendon and the degree of rotation. The biomechanical effect of tenotomy will vary depending on the incision level, which directly depends on the location and rotation of the subtendons, as demonstrated in our study.

The possible role of the plantaris tendon in the calcaneal tendinopathy has been studied previously [7, 19]. The plantaris tendon is close or directly related to the medial outline of the calcaneal tendon, mostly in types 1 and 2 according to Olewnik et al. [19], as revealed in our study and reported previously in adults [8, 19, 32]. In our material, types 1 and 2 constitute approximately 65% of the plantaris tendon variants, similar to previous studies [19]. The difference lies in the percentage of each kind of plantaris. In our material, both types occur in quite similar amounts, whereas according to Olewnik et al. [19] type 1 is dominant. It is difficult to explain the difference, but it could result from developmental factors or be related to the group selection.

The proximity of the plantaris tendon to the calcaneal tendon, even in the second trimester, may indicate that the previously described role of the plantaris tendon in the calcaneal tendon tendinopathy [19, 32] may also have a developmental background. However, we did not find studies of the correlation between the particular plantaris variants and the occurrence of the calcaneal tendinopathy. Even in the early stages of development, the calcaneal tendon and plantar fascia showed a mutual attachment to the calcaneal perichondrium, which could contribute to form a functional mechanical and developmental unit [28]. According to previous observations, dysfunction of the calcaneal tendon may cause the development of the plantar fascia pathology, and vice versa [31, 32]. Rehabilitation of the calcaneal tendon may be useful in the treatment of plantar fasciitis. We did not reveal the anatomical connection of the plantaris tendon and plantar fascia.

The limitations of our study are its postmortem character and the lack of information on the cause of intrauterine death, the exact age of the fetuses and the tibial rotation angle.

The structure of the calcaneal tendon in the human fetus in the second trimester is similar to that in the adult calcaneal tendon and is made up of twisted subtendons. This means that the calcaneal tendon development occurs during the first trimester. Research on material from earlier stages of development than 13 weeks’ gestation is necessary to follow development of the calcaneal tendon.

## Data Availability

Data transparency: Yes.

## Code availability

Software application or custom code: Yes.
